# Pretransplant serum levels of endothelial cell activation markers are associated with graft loss and mortality after kidney transplantation

**DOI:** 10.1111/sji.13225

**Published:** 2022-11-08

**Authors:** Kit Peiter Lund, Frank Eriksson, Bente Klarlund Pedersen, Søren Schwartz Sørensen, Helle Bruunsgaard

**Affiliations:** ^1^ Department of Clinical Immunology 7631 University Hospital of Copenhagen – Rigshospitalet Copenhagen Denmark; ^2^ Section of Biostatistics, Department of Public Health University of Copenhagen Copenhagen Denmark; ^3^ Center of Inflammation and Metabolism and Centre for Physical Activity Research University Hospital of Copenhagen ‐ Rigshospitalet Copenhagen Denmark; ^4^ Department of Clinical Medicine University of Copenhagen Copenhagen Denmark; ^5^ Department of Nephrology P University Hospital of Copenhagen – Rigshospitalet Copenhagen Denmark

**Keywords:** adhesion molecules, inflammation, solid organ transplantation, transplantation

## Abstract

Long‐term allograft survival remains a challenge in kidney transplantation. In this study, we aimed to identify biomarkers for potentially modifiable pathways involved in the outcome of kidney transplantation. We tested the hypothesis that a pre‐existing systemic environment with endothelial cell activation in the recipient is associated with the outcome after kidney transplantation. In a retrospective study cohort of 611 kidney transplanted patients, we investigated associations between serum levels of soluble intercellular adhesion molecule‐1 (sICAM‐1) and soluble vascular cell adhesion molecule‐1 (sVCAM‐1) before transplantation and delayed graft function, acute rejection, graft loss and mortality after transplantation. We adjusted associations for age, sex, preformed donor‐specific antibodies (DSA), pretransplant diabetes, cardiovascular disease and dialysis. Additionally, we investigated if associations between endothelial cell activation markers and outcomes differed in recipients with and without preformed DSA. Serum levels of endothelial cell activation markers were associated with delayed graft function and mortality but not with rejection. Additionally, high levels of sICAM‐1 were associated with graft loss. Associations were most pronounced in recipients without DSA, adjusted for potential confounders. Data suggest that endothelial cell activation at the time of transplantation is associated with graft loss and mortality after kidney transplantation, especially in transplant candidates without preformed DSA.

## 1 INTRODUCTION

End‐stage kidney failure is responsible for high mortality worldwide, and kidney transplantation is the first treatment of choice for suitable recipients.^1^ Limiting factors are shortage of available organs for transplantation combined with the lack of improvement in the long‐term prognosis for kidney transplantation over the past decades^2^

Chronic rejection defines decreasing function of the transplanted kidney (allograft) and has become the primary cause of allograft loss.^3^ Today, chronic rejection is considered a process beginning any time after transplantation and progressively leading to chronic allograft dysfunction and loss.^4^ The dominant theory is that the leading cause of rejection is microvascular inflammation triggered by antibodies against (non‐self) alloantigens.^4^ Donor‐specific antibodies (DSA) are allogenic antibodies directed towards non‐self HLA molecules of the donor. Preformed DSAs are present already before transplantation whereas so‐called de novo DSAs can emerge at any time after transplantation.^4^, ^5^ Both preformed DSA and de novo DSA are known to play a significant role and to be among the strongest predictors of subsequent short‐term and long‐term graft failure in kidney transplantation.^4^, ^5^ A few years ago, it was described that among kidney allograft recipients who develop de novo DSA, a subset of recipients exhibits severe arteriosclerosis in the allograft.^6^ These recipients also have an increased risk of experiencing major adverse cardiovascular events (MACE) and high mortality.^6^ The latter observation suggests that inflammation in the graft may result in systemic inflammation that accelerates vascular inflammation and atherosclerosis in the native arteries of the recipient. Alternatively, the development of alloreactivity is a consequence of a genetic or environmental predisposition in the recipient to develop vascular inflammation and atherosclerosis^7^

End‐stage kidney failure is associated with chronic systemic inflammation that predisposes individuals to insulin resistance, dyslipidaemia, endothelial dysfunction and accelerated atherosclerosis.^8^ Proinflammatory cytokines such as tumour necrosis factor (TNF)‐α and interleukin(IL)‐1 induce endothelial cell activation as well as initiate IL‐6 production, which stimulates the liver production of acute‐phase reactants, such as C‐reactive protein (CRP).^9^ Endothelial cell activation includes increased expression of HLA antigens and cellular adhesion molecules (CAMs), which are central mechanisms in vascular inflammation, atherosclerosis, MACE and allograft rejection in solid organ transplantation.^9^ Proinflammatory cytokines, bacterial endotoxin and oxidized low‐density lipoprotein induce increased endothelial cell expression of CAMs.^10^, ^11^, ^12^ Intercellular CAM (ICAM)‐1 and vascular CAM (VCAM)‐1 contribute to the control of the adhesion and the extravasation of leucocytes on the endothelium.^11^ This recruitment of blood leukocytes is considered an early step in the process of atherosclerosis.^13^ Soluble forms of ICAM‐1 (sICAM‐1) and VCAM‐1 (sVCAM‐1) are detected in serum^10^ and they are shed from endothelial cells in response to cytokine activation.^14^ There is a positive correlation between the endothelial expression of ICAM‐1/VCAM‐1 and levels of sICAM‐1/sVCAM‐1 in both dose–response and time–response experiments following TNF‐α in vitro stimulation, suggesting sCAMs mirror endothelial cell activation.^15^ Levels of sCAMs correlate with the extent of underlying atherosclerosis, for example^16^, ^17^ suggesting sCAMs as biomarkers of vascular inflammation, endothelial dysfunction and the vascular atherosclerotic burden.^18^, ^19^, ^20^ In type 1 diabetes sCAMs are associated with microalbuminuria and nephropathy^21^ and sICAM‐1, but not hsCRP, IL‐6 or TNF‐α, is associated with progressive nephropathy, suggesting sICAM‐1 to be a mediator of progressive microvascular disease.^22^ Enhanced systemic levels of sCAMs precede MACE in patients with cardiovascular disease^20^, ^23^ and is associated with increased all‐cause mortality in patient with medium to high risk of cardiovascular disease^24^ as well as in patients with type II diabetes and microalbuminuria.^25^ In a study of kidney graft biopsies from patients with chronic rejection the expression of ICAM‐1 and VCAM‐1 was increased.^26^ Additionally, genetic polymorphisms in endothelial cell activation markers have been linked to acute rejections in kidney transplantation.^27^ Moreover, systemic inflammation is accompanied by immune activation that may promote the production of de novo DSA. For example, among many effects, IL‐6 is essential for B cell differentiation, antibody production and the generation of long‐lived plasma cells^28^, ^29^

Previously, we have shown that pre‐existing chronic systemic inflammation at the time of transplantation predicts delayed graft function and all‐cause mortality in kidney transplanted recipients.^30^ In the present study, our ambition was to explore mechanisms that link systemic inflammation at the time of transplantation with mortality in the following years. We investigated if soluble adhesion molecules (sICAM‐1 and sVCAM‐1) before transplantation were associated with delayed graft function (DGF), acute rejection within the first year, graft loss and mortality after kidney transplantation. Additionally, we investigated if associations of sCAMs and outcomes were different in recipients with and without preformed DSA. The rationale was that it has been reported that kidney transplanted recipients with HLA antibodies exhibit a ‘proinflammatory’ phenotype that may accelerate vessel disease.^6^ The study focus was on factors and mechanisms in recipients that affect the outcome after transplantation. The overall vision was to identify new useful biomarkers in potentially modifiable pathways for future treatments in kidney transplant recipients

## 2 MATERIALS AND METHODS

### 2.1 Study cohort

We included all recipients who underwent kidney transplantation from a deceased or living donor at Copenhagen kidney transplantation centres (Herlev Hospital and Rigshospitalet, University Hospital of Copenhagen) from 1 January 2009 to 31 December 2015 (n = 632).

Thirteen recipients were excluded from the study due to missing or too little pretransplant serum for research purposes. Eight recipients were transplanted twice in the inclusion period, and only the first transplantation was included in the study. Consequently, a total number of 611 recipients were included in this retrospective study. Recipients were routinely evaluated by complement‐dependent cytotoxicity (CDC) screen prior to transplantation. All recipients had negative prospective B cell CDC cross‐matches with dithiothreitol‐treated recipient serum. The two transplantation centres had similar immunosuppressive induction protocols as well as similar treatments for acute rejection episodes. No additional immunosuppression was given to recipients with preformed DSA, as the historical algorithm in the transplantation centres did not include Luminex‐based DSA status at the time of transplantation during the inclusion period of the study cohort

Included recipients were tracked until July 21st, 2020, resulting in a median follow‐up of 8.3 years, ranging from 4.6 to 11.5 years. The primary outcome of the study was all‐cause mortality. Secondary outcomes were acute rejection during the first year after transplantation, DGF as an early marker of graft function, and graft loss within the follow‐up time, representing the long‐term outcome

The cohort has been previously described.^30^, ^31^ In brief, data collection on recipient age, recipient sex and transplant number were obtained from the Scandiatransplant Database. We acquired data on acute rejections, graft loss and death from the Danish Nephrology Registry. We defined MACE as major thromboembolisms in heart, brain or visceral arteries and obtained data from The Danish National Registry 10th revision. DGF was defined as the requirement for dialysis in the first post‐operative week

### 2.2 Pretransplant HLA‐antibody evaluation

Recipient serum samples obtained at the time of transplantation were retrospectively screened for preformed anti‐HLA antibodies by LABScreen™ Mixed (One Lambda, Maryland, US). Positive samples were further analysed by LABScreen™ Single Antigen (One Lambda, Maryland, US) with positivity defined as mean fluorescence intensity (MFI) values ≥1000. The DSA variable was dichotomized into recipients with or without preformed DSA, defined as recipient reactions with single antigen beads, which carried one or more of the donor's HLA‐A, ‐B, ‐Cw, ‐DR and ‐DQ antigens

### 2.3 Pretransplant soluble endothelial cell activation markers

Recipient serum concentrations of sICAM‐1 and sVCAM‐1 were retrospectively analysed by MSD® V‐PLEX Custom Human Biomarkers assay (Meso Scale Diagnostics, Maryland, US) according to the manufacturer's instructions. The lower detection limits were 1.03 pg/mL (dynamic range 1.03‐42 708 pg/mL) for sICAM‐1 and 6.00 pg/mL (dynamic range 6.00‐31 960 pg/mL) for sVCAM‐1. All samples were within the detection range. In our laboratory, the inter‐assay coefficient of variation (CV) was 29% for sICAM‐1 and 25% for sVCAM‐1. All samples were run as duplicates, and the mean values were calculated. Deviations in duplicates had to be <20% to accept the analysis. Serum concentrations of high‐sensitive CRP (hsCRP) and inflammatory cytokines at the time of transplantation were measured as previously described by Lund et al., 2019.^30^

### 2.4 Statistics

Statistical analyses were performed using IBM SPSS Statistics 22 and R.^32^ Duration of follow‐up was calculated as the time from inclusion (transplantation) until data retrieval on July 21st, 2020. We compared categorical variables by Chi‐square test and continuous variables between independent groups by Wilcoxon's rank sum test. Spearman's correlation analysis was used for assessing monotone relationships between continuous variables. Survival and cumulative incidence curves were estimated by the Kaplan–Meier and Aalen‐Johansen estimators, and compared by log‐rank and Grey's tests, respectively. Cox regression was used to investigate associations between pretransplantation DSA and levels of soluble endothelial cell markers (explanatory variables) and acute rejection, graft loss and mortality hazards (outcomes). When analysing acute rejection and graft loss, death was treated as a competing risk.

Cox regression models were adjusted for confounders defined as variables associated with endothelial cell activation and having an independent effect on the outcome variable. Model 1 was adjusted for age and sex. Model 2 was adjusted for preformed DSA as well as age and sex. Model 3 was additionally adjusted for pretransplant diabetes mellitus (DM), MACE and dialysis status. Pretransplant DM, pretransplant MACE and pretransplant dialysis are potential confounders or perhaps intermediate factors. Therefore, we chose the strategy to perform statistical analyses both with and without adjustment for these covariates.

We tested also if associations between endothelial cell activation markers and outcome variables differed in recipients with and without preformed DSA by an interaction in model 4 and 5 with and without adjustments for pretransplant DM, pretransplant MACE and pretransplant dialysis. Missing information on pretransplant dialysis and pretransplant DM was handled by multiple imputation (treating death as a competing risk and using 25 imputations) with the R package SMCFCS.^33^ Endothelial cell activation markers were included untransformed in all statistical analyses. All hazard ratios (HR) are for 100 ng/mL difference in sICAM‐1 and sVCAM‐1. Model diagnostics assessed the validity of the proportional hazards assumption and covariates' functional form based on cumulative sums of martingale residuals.^34^ Binary logistic regression analyses were used to test for associations between CAMs and DGF. *P* ≤ .05 were considered to indicate statistical significance in all analyses.

### 2.5 Ethical considerations

This study was approved by the regional ethical authority (code number H‐16028690). The research biobank and the research database were approved by the Danish Protection Agency (RH‐2016‐240, I‐suite no: 04840).

## 3 RESULTS

### 3.1 Characteristics of the study population

Cohort characteristics are summarized in Table 1. The median age of recipients was 48 years, with a range of 1 to 83 years of age at transplantation. Recipients in the cohort were predominantly male (62%) receiving their first kidney transplantation (85%). A minor fraction of the recipients had a medical history of MACE (13%). Data on pretransplant DM were available for 590 recipients, of whom 15% were diagnosed with DM before kidney transplantation. Data on pretransplant dialysis was available for 590 recipients, of whom the majority (87%) were dependent of dialysis prior to transplantation and corresponding 13% underwent pre‐emptive transplantation.

**TABLE 1:** Descriptive cohort characteristics of recipient factors including demographic variables, comorbidity, transplantation data and markers of vascular inflammation

Recipients	All (n = 611)	No preformed DSA (n = 460)	Preformed DSA (n = 151)	*P*‐value
Patient characteristics				
Age at transplantation (years)—Median (25%‐75%)	48 (36‐59)	49 (37‐59)	46 (35‐58)	.15
Sex—No. (%)				
Men	381 (62%)	315 (68%)	66 (44%)	**<.001**
Women	230 (38%)	145 (32%)	85 (56%)	
Pretransplant DM^a^—No. (%)	87 (15%)	72 (16%)	15 (10%)	.09
Pretransplant MACE—No. (%)	77 (13%)	56 (12%)	21 (14%)	.68
Pretransplant dialysis^b^—No. (%)	514 (87%)	384 (87%)	130 (88%)	.87
Transplantation characteristics				
Transplantation history—No. (%)				
Fist time transplantation	522 (85%)	417 (91%)	105 (69%)	**<.001**
Retransplantation	89 (15%)	43 (9%)	46 (31%)	
Donor type—No. (%)				
Deceased donor	380 (62%)	282 (61)	98 (65)	.49
Living donor	231 (38%)	178 (39%)	53 (35%)	
Donor age (years)—Median (25%‐75%)	52 (41‐61)	51 (41‐61)	54 (42‐62)	.33
ABO incompatibility—No. (%)	54 (9%)	40 (9%)	14 (9%)	.96
HLA‐A/B/DR mismatches—Median (25%‐75%)	3 (2‐4)	3 (2‐4)	3 (2‐4)	.81
Cold ischemia time/Living donor^c^ (hours)—Median (25%‐75%)	2.8 (2.2‐3.4)	2.8 (2.2‐3.4)	2.8 (2.3‐3.5)	.78
Cold ischemia time/Deceased donor^c^ (hours)—Median (25%‐75%)	18 (14‐22)	18 (13‐22)	19 (15‐22)	.13
Vascular inflammation biomarkers				
sICAM‐1 (100 ng/mL)—Median (25%‐75%)	5.16 (4.13‐6.49)	5.13 (4.12‐6.37)	5.27 (4.21‐6.72)	.21
sVCAM‐1 (100 ng/mL) – Median (25%‐75%)	11.90 (9.65‐14.44)	11.71 (9.53‐14.11)	12.59 (10.24‐15.22)	**.01**

*Note*: For continuous variables medians (25%‐75%) are shown. For categorical variable No.(%) is shown. Chi‐square test was used for the comparison of categorical variables. Wilcoxon's rank sum test was used for the comparison of continuous variables. The *P*‐value refers to comparison between recipients with and without preformed DSA. *P* < .05 was considered significant and marked to be bold.

Abbreviations: DM, diabetes mellitus; DSA, donor‐specific antibodies; MACE, major adverse cardiovascular events; No., number; sICAM‐1, soluble intercellular adhesion molecule 1; sVCAM‐1, soluble vascular cell adhesion protein 1.

^a^ Data on pretransplant DM was available for 590 recipients (missing for 21 recipients), corresponding to 96.6% of the included recipients.

^b^ Data for pretransplant dialysis was available for 590 recipients (missing for 21 recipients), corresponding to 96.6% of the included recipients.

^c^ Data for cold ischemia time was available for 579 recipients (missing for 32 recipients), corresponding to 94.8% of the included recipients.

One‐hundred‐fifty recipients experienced an acute rejection during the first year, 93 recipients died, and 92 lost their graft during the follow‐up period.

Fifty‐nine percent (N = 361) of the recipients were sensitized with HLA alloantibodies directed towards non‐self HLA molecules at the time of transplantation, and 25% (N = 151) had preformed DSA directed towards non‐self HLA molecules in the donor. Accordingly, 42% (151/361) of HLA allo‐immunized recipients presented preformed DSA. Among patients with preformed DSA the frequency of women was higher compared to men and the frequency of previously transplanted patients was higher compared to first‐time transplants (Table 1). Presence of preformed DSA was associated with acute rejection (HR: 1.49 (95% confidence interval [CI]: 1.04‐2.14), *P* = .03) as well as graft loss (HR: 1.88 [95%CI: 1.21‐2.94], *P* = .005) adjusted for age and sex, where model control indicated this was most pronounced during the first 3‐4 years after transplantation. We did not find a statistically significant association between preformed DSA and mortality (HR: 1.25 [95%CI: 0.75‐2.09], *P* = .40).

### 3.2 Cohort characteristics concerning serum levels of sICAM‐1 and sVCAM‐1

Serum levels of sICAM‐1 and sVCAM‐1 were intercorrelated (*r*_s_ = 0.60, *P* < .001), and both endothelial cell activation markers were correlated with serum concentrations of TNF‐α, IL‐6 and hsCRP (Table 2). Pretransplant dialysis was associated with higher levels of both sICAM‐1 and sVCAM‐1. No difference was found in serum concentrations of endothelial cell activation markers between groups concerning age, sex, pre‐existing DM, pre‐existing MACE or first transplantation versus subsequent transplantation. Pretransplant DSA was associated with sVCAM‐1.

**TABLE 2:** Associations between recipient factors, preformed donor‐specific antibodies and markers of systemic inflammation in relation to vascular inflammation markers

Recipients	No.	sICAM‐1 (100 ng/mL)	*P*‐value	sVCAM‐1 (100 ng/mL)	*P*‐value
Age at transplantation	611	*r*_s_ = 0.06	.14	*r*_s_ = 0.00	.96
Sex					
Men	381	5.11 (4.03‐6.68)	.64	11.66 (9.53‐14.51)	.69
Women	230	5.17 (4.26‐6.39)		11.95 (9.71‐14.43)	
Transplantion history					
First transplantation	522	5.17 (4.16‐6.51)	.27	11.71 (9.65‐14.27)	.17
Retransplantation	89	5.02 (4.07‐6.03)		12.74 (10.05‐14.90)	
Pretransplant DM^a^					
No	503	5.14 (4.11‐6.49)	.66	11.70 (9.61‐14.30)	.40
Yes	87	5.23 (4.33‐6.45)		12.26 (9.63‐15.42)	
Pretransplant dialysis^b^					
No	76	4.86 (3.89‐5.69)	**.03**	9.98 (7.80‐12.70)	**<.001**
Yes	514	5.21 (4.15‐6.66)		12.00 (9.93‐14.53)	
Pretransplant MACE					
No	534	5.14 (4.11‐6.48)	.07	11.78 (9.60‐14.43)	.21
Yes	77	5.35 (4.71‐6.96)		12.47 (10.62‐14.48)	
Preformed DSA					
No	460	5.13 (4.12‐6.35)	.21	11.71 (9.54‐14.09)	**.01**
Yes	151	5.27 (4.21‐6.71)		12.59 (10.32‐15.17)	
Inflammatory biomarkers					
TNF‐α	611	*r*_s_ = 0.39	**<.001**	*r*_s_ = 0.38	**<.001**
IL‐6	611	*r*_s_ = 0.27	**<.001**	*r*_s_ = 0.20	**<.001**
hsCRP	611	*r*_s_ = 0.25	**<.001**	*r*_s_ = 0.11	**.006**

*Note*: Median (25%‐75%) is shown for independent groups. Wilcoxon's rank sum test was used for the comparison of markers of endothelial activation between independent groups. *r*_s_, Spearman's correlation was used for assessing monotone relationships between continuous variables. *P* < .05 was considered significant and marked to be bold.

Abbreviations: cPRA, calculated panel reactive antigens; DM, diabetes mellitus; HLA, human leukocyte antigen; hsCRP, high‐sensitivity C‐reactive protein; IL, interleukin; MACE, major adverse cardiovascular events; No., number; sICAM‐1, soluble intercellular adhesion molecule 1; sVCAM‐1, soluble vascular cell adhesion protein 1; TNF, tumour necrosis factor.

^a^ Data on pretransplant DM was available for 590 recipients, corresponding to 96.6% of the included recipients (missing data for 21 recipients).

^b^ Data on pretransplant dialysis was available for 590 recipients, corresponding to 96.9% of the included recipients (missing data for 21 recipients).

### 3.3 Associations between pretransplant soluble intercellular adhesion molecules and all‐cause mortality

**FIGURE 1:**
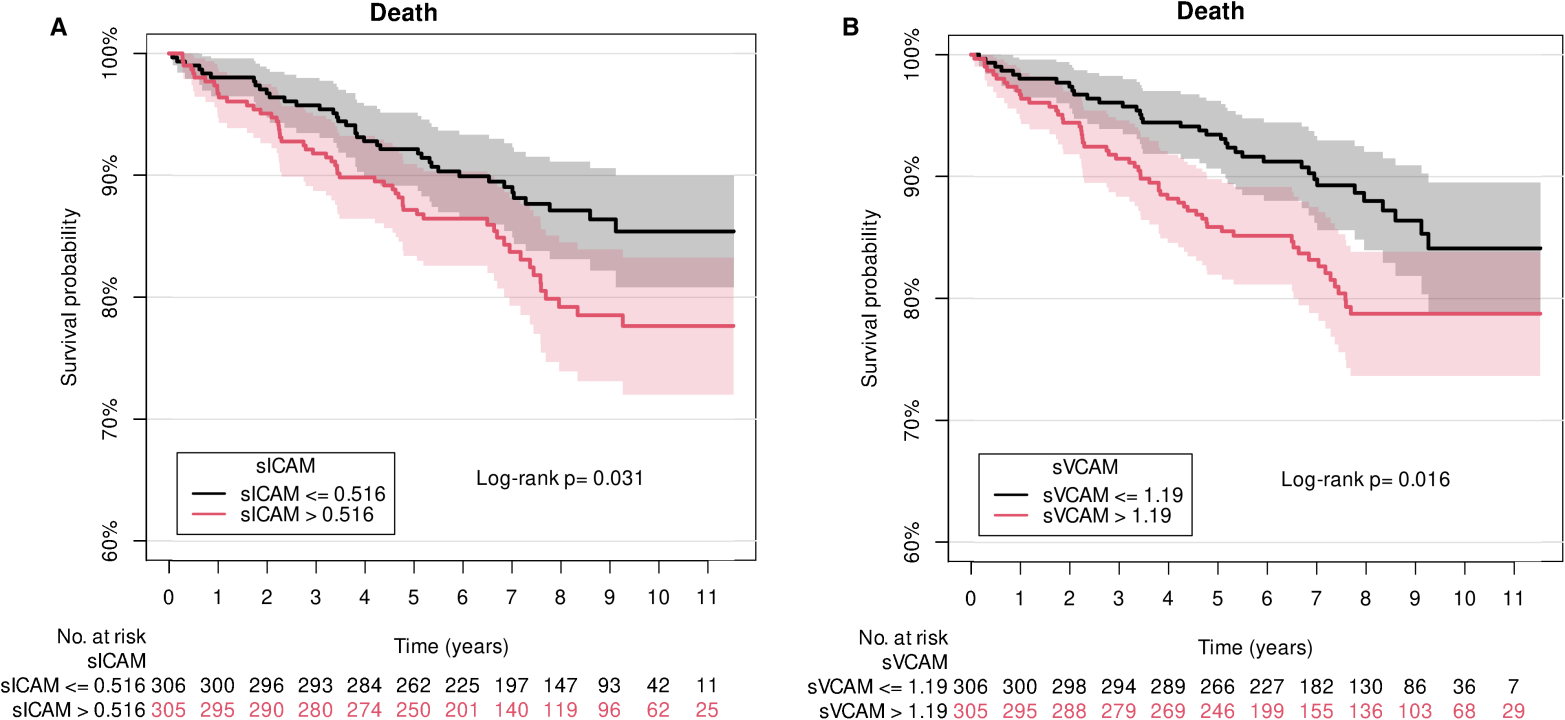
Kaplan–Meier curves for all‐cause mortality in recipients with low and high levels of endothelial cell activation markers at the time of transplantation. Kaplan–Meier curves regarding all‐cause mortality and endothelial cell activation markers: sICAM‐1 (A) and sVCAM‐1 (B). Recipients are divided into two groups based on the median level of markers: lowest 50% (black line) and highest 50% (red line). Below the graphs are numbers at risk. 95% confidence intervals are shown. sICAM‐1: soluble intercellular adhesion molecule 1; sVCAM‐1: soluble vascular cell adhesion protein 1.

Serum levels of sICAM‐1 and sVCAM‐1 at the time of transplantation were both associated with all‐cause mortality adjusted for age and sex in the whole cohort of kidney transplanted recipients (Table 3A and Figure 1A,B). HR of associations were almost unaffected after further adjustment for DSA in model 2. When pretransplant DM, MACE and dialysis status were also added the HR of sICAM‐1 was almost unaltered and the association with mortality persisted (Model 3). In contrast, the HR of sVCAM‐1 decreased slightly and did not reach statistical significance (*P* = .15).

**TABLE 3:** Associations between markers of vascular inflammation, preformed DSA and outcomes after kidney transplantation

	No. of recipients	sICAM‐1100 ng/mL HR (95%CI), *P*‐value	sVCAM‐1100 ng/mL HR (95%CI), *P*‐value
(A) All‐cause mortality			
Model 1	611	1.16 (1.05‐1.28), **.003**	1.06 (1.02‐1.11), **.009**
Model 2	611	1.15 (1.05‐1.27), **.004**	1.06 (1.01‐1.11), **.01**
Model 3	611	1.15 (1.04‐1.28), **.008**	1.04 (0.99‐1.09), .15
Model 4 – Test for interaction		*P* = .25	*P* = .25
No preformed DSA	460 (75)	1.21 (1.07‐1.36), **.002**	1.08 (1.02‐1.14), **.005**
Preformed DSA	151 (25)	1.06 (0.88‐1.28), .54	1.01 (0.92‐1.12), .83
Model 5 – Test for interaction		*P* = .38	*P* = .48
No preformed DSA	460 (75)	1.19 (1.05‐1.35), **.007**	1.05 (0.99‐1.11), .10
Preformed DSA	151 (25)	1.07 (0.88‐1.30), .50	1.00 (0.91‐1.11), .94
(B) Graft loss			
Model 1	611	1.10 (1.00‐1.22), **.05**	1.03 (0.98‐1.07), .25
Model 2	611	1.09 (0.99‐1.20), .09	1.02 (0.97‐1.07), .45
Model 3	611	1.08 (0.98‐1.19), .11	1.02 (0.97‐1.06), .53
Model 4—Test for interaction		*P* = .16	*P* = .21
No preformed DSA	460 (75)	1.16 (1.02‐1.33), **.02**	1.04 (0.98‐1.11), .15
Preformed DSA	151 (25)	1.00 (0.85‐1.18), .97	0.98 (0.91‐1.06), .64
Model 5—Test for interaction		*P* = .18	*P* = .19
No preformed DSA	460 (75)	1.16 (1.01‐1.32), **.03**	1.04 (0.98‐1.11), .16
Preformed DSA	151 (25)	1.00 (0.86‐1.18), .97	0.98 (0.90‐1.06), .55
(C) Acute rejection			
Model 1	611	0.99 (0.91‐1.07), .77	0.99 (0.96‐1.03), .76
Model 2	611	0.98 (0.90‐1.07), .66	0.99 (0.95‐1.03), .60
Model 3	611	0.98 (0.90‐1.07), .64	0.99 (0.95‐1.03), .54
Model 4—Test for interaction		*P* = .70	*P* = .96
No preformed DSA	460 (75)	0.97 (0.87‐1.08), .56	0.99 (0.94‐1.04), .69
Preformed DSA	151 (25)	1.00 (0.88‐1.13), 1.00	0.99 (0.92‐1.06), .72
Model 5—Test for interaction		*P* = .63	*P* = .93
No preformed DSA	460 (75)	0.96 (0.86‐1.08), .51	0.99 (0.94‐1.04), .59
Preformed DSA	151 (25)	1.00 (0.89‐1.14), .96	0.99 (0.93‐1.06), .77
(D) Delayed graft function			
Model 1	513	1.10 (1.00‐1.21), **.05**	1.08 (1.03‐1.13), **.001**
Model 2	513	1.10 (1.00‐1.21), .06	1.07 (1.03‐1.12), **.002**
Model 3	513	1.10 (1.00‐1.21), .06	1.07 (1.03‐1.12), **.002**
Model 4—Test for interaction		*P* = .11	***P* = .01**
No preformed DSA	383	1.17 (1.03‐1.32), **.01**	1.12 (1.06‐1.18), **<.001**
Preformed DSA	130	0.99 (0.84‐1.16), .88	0.99 (0.92‐1.07), .90
Model 5—Test for interaction		*P* = .10	***P* = .02**
No preformed DSA	383	1.17 (1.03‐1.32), **.01**	1.12 (1.06‐1.18), **<.001**
Preformed DSA	130	0.98 (0.83‐1.16), .82	0.99 (0.91‐1.07), .85

*Note*: All HRs and ORs are for 100 ng/mL difference in sICAM‐1 and sVCAM‐1. (A‐C) Cox regression models. Model 1: Adjusted for age and sex. Model 2: Adjusted for age, sex and preformed DSA (no/yes). Model 3: Adjusted for age, sex, preformed DSA, pretransplant DM, pretransplant MACE and pretransplant dialysis. Model 4: Model with interaction between serum levels of sICAM‐1/sVCAM‐1 and preformed DSA (no/yes) adjusted for age and sex. Model 5: Model with interaction between serum levels of sICAM‐1/sVCAM‐1 and preformed DSA (no/yes) adjusted for age, sex, pretransplant DM, pretransplant MACE and pretransplant dialysis. In Cox regression models, missing information on pretransplant dialysis and pretransplant DM was handled by multiple imputation (treating death as a competing risk and using 25 imputations) with the R package SMCFCS. (D) Logistic regression analysis. Models 1‐5 are adjusted for the same variable as Cox regression model but without pretransplant dialysis in model 3 and model 5 as only patients with pretransplant dialysis are included in analyses. *P* < .05 was considered significant and marked to be bold.

Abbreviations: CI, confidence interval; DSA, donor‐specific HLA antibodies; HR, hazard ratio; MACE, major adverse cardiovascular events; No., number; sICAM‐1, soluble intercellular adhesion molecule 1; sVCAM‐1, soluble vascular cell adhesion protein 1.

To investigate if associations between endothelial cell activation markers and outcomes differed in recipients with and without preformed DSA, an interaction between endothelial cell activation markers and DSA status was introduced in model 4. In this model there was a significant association of both sICAM‐1 and sVCAM‐1 with mortality in recipients without DSA adjusted for sex and age. When DM, MACE and dialysis status were also added to the model (model 5), HR of sICAM‐1 was almost unaffected. In contrast, the HR of sVCAM‐1 declined slightly and did not reach statistical significance (*P* = .10). In recipients with preformed DSA, associations between both endothelial cell activation markers and mortality had a 95%CI that included 1 and did not reach significance. Moreover, there was no significant interaction between endothelial cell activation markers and DSA status (Model 4 and model 5). Consequently, the null hypothesis that the association between vascular inflammation and graft loss was the same in recipients with and without DSA could not be rejected.

Finally, an extended analysis was performed to investigate associations between markers of endothelial cell activation and mortality adjusted for a wide range of traditional risk factors for complications after transplantation, namely deceased donor, log_2_‐transformed cold ischemia time, ABO incompatibility, HLA‐A/B/DR mismatches, together with preformed DSA, recipient age and recipient sex. The model included an interaction between donation type and cold ischemia time. In these models pretransplant levels of both endothelial cell activation markers were associated with mortality: sICAM‐1 (HR: 1.15 [95%CI: 1.04‐1.28], *P* = .006) and sVCAM‐1 (HR: 1.06 [95%CI: 1.01‐1.11], *P* = .02).

### 3.4 Associations between pretransplant soluble intercellular adhesion molecules and graft loss

**FIGURE 2:**
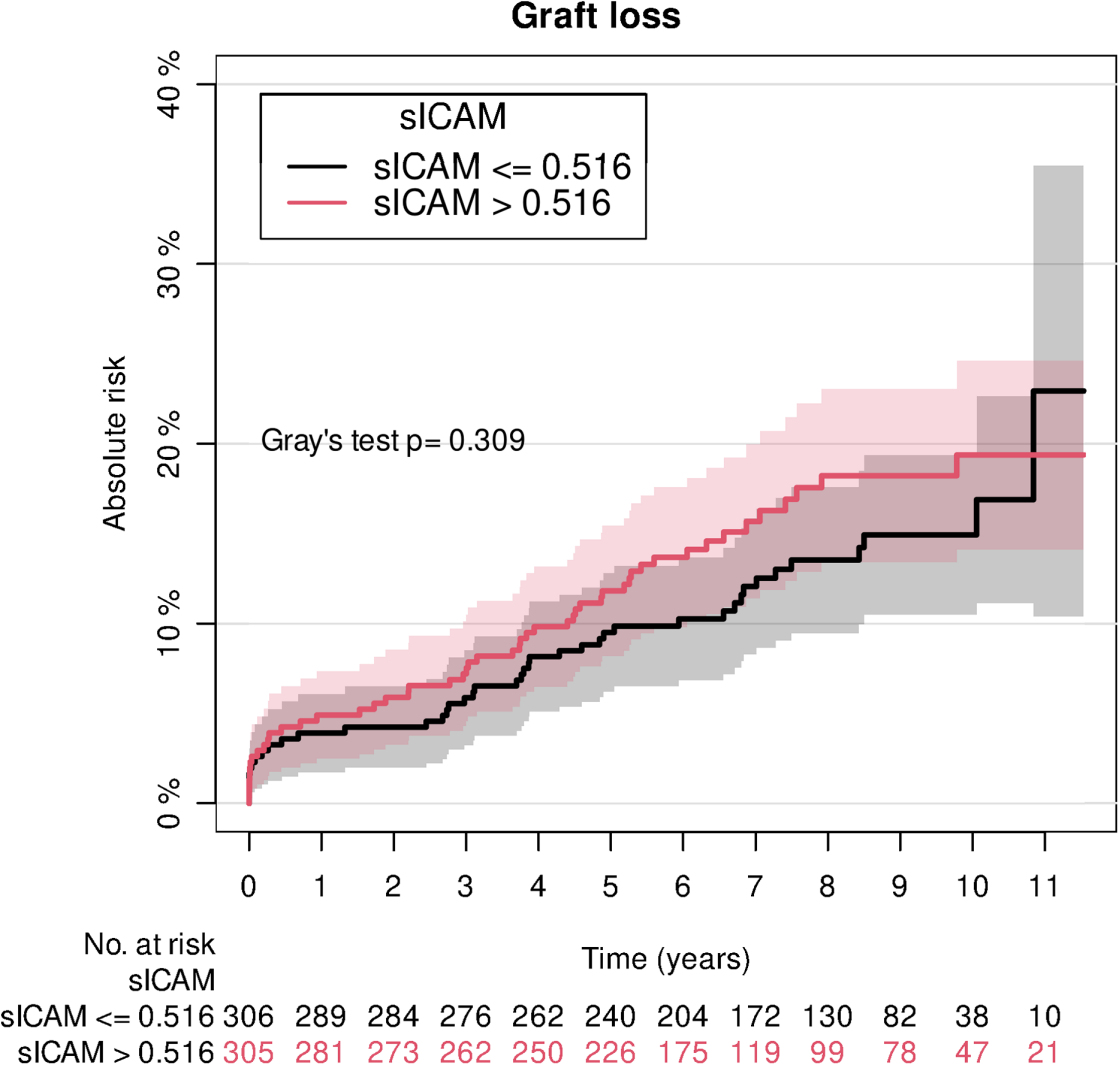
Cumulative incidence for graft loss, according to levels of soluble endothelial cell activation markers at the time of transplantation. Curves of cumulative incidence regarding graft loss and concentrations of sICAM‐1. Recipients are divided into two groups based on the median level of markers: lowest 50% (black line) and highest 50% (red line). Death was treated as a competing risk in the analysis. Below the graphs are numbers at risk. 95% confidence intervals are shown. sICAM‐1: soluble intercellular adhesion molecule 1.

The serum level of sICAM‐1 was significantly associated with graft loss in a Cox regression analysis, adjusted for age and sex (Table 3B, model 1 and Figure 2). When DSA was added in model 2, the HR of sICAM‐1 was slightly diminished, and the change of 95%CI was minimal, but the P‐value was above the established significance level. When an interaction between DSA and sICAM‐1 was included in model 4, sICAM‐1 was significantly associated with graft loss in recipients without preformed DSA, whereas the test for association did not reach statistical significance for recipients without preformed DSA. Moreover, there was no significant interaction between DSA status and sICAM‐1. (Table 3B, model 4). Accordingly, the null hypothesis, that the association between sICAM‐1 and graft loss was the same in recipients with and without DSA, could not be rejected. The analysis showed that recipients without preformed DSA had 16% increased hazard of graft loss per 100 ng/mL higher sICAM‐1 at the time of transplantation. There was no significant association between sVCAM‐1 and graft loss in models 1‐4 (Table 3B). In model 5 we adjusted for additional potential confounders, namely DM, MACE, dialysis with only minor changes compared to model 4.

In an extended model including traditional risk factors for complications after transplantation (deceased donor, log2‐transformed cold ischemia time according to living/ deceased donor, ABO incompatibility, HLA‐A/B/DR mismatches, preformed DSA), alongside recipient age and sex there was no association between endothelial cell activation markers and graft loss. For sICAM‐1 HR: 1.07 (95%CI: 0.96‐1.19), *P* = .20 and for sVCAM‐1 HR: 1.01 (95%CI: 0.96‐1.06), *P* = .62.

DGF is a risk factor of early graft loss.^35^ We tested if vascular inflammation in the transplant candidate with and without DSA was associated with DGF in logistic regression models. Patients were only included in the analyses if they had received pretransplant dialysis as DGF was defined as the requirement for dialysis in the first post‐operative week (N = 514, Table 1). Among these 514 patients, 146 patients (28%) needed dialyses during the first week. The 146 patients also included 12 patients who never obtained graft function. One patient had missing data on DGF and was, therefore, excluded from analyses. Both sICAM‐1 and sVCAM‐1 were associated with DGF or no graft function al all adjusted for sex and age (Table 3D). The association persisted for sVCAM‐1 after further adjustments for DSA, pretransplant MACE and pretransplant DM whereas it did exactly not reach significance (*P* = .06) for sICAM‐1 although HR and 95%CI were minimal affected. When analyses were stratified by DSA both ICAM‐1 and sVCAM‐1 were associated with DGF in patients without DSA but not in patients with DSA. There was a significant interaction between DSA and sVCAM‐1, supporting the association between sVCAM‐1 and DGF was different in recipients with and without DSA whereas there was no significant interaction between DSA and sICAM‐1

### 3.5 Associations between pretransplant soluble intercellular adhesion molecules and acute rejection

**FIGURE 3:**
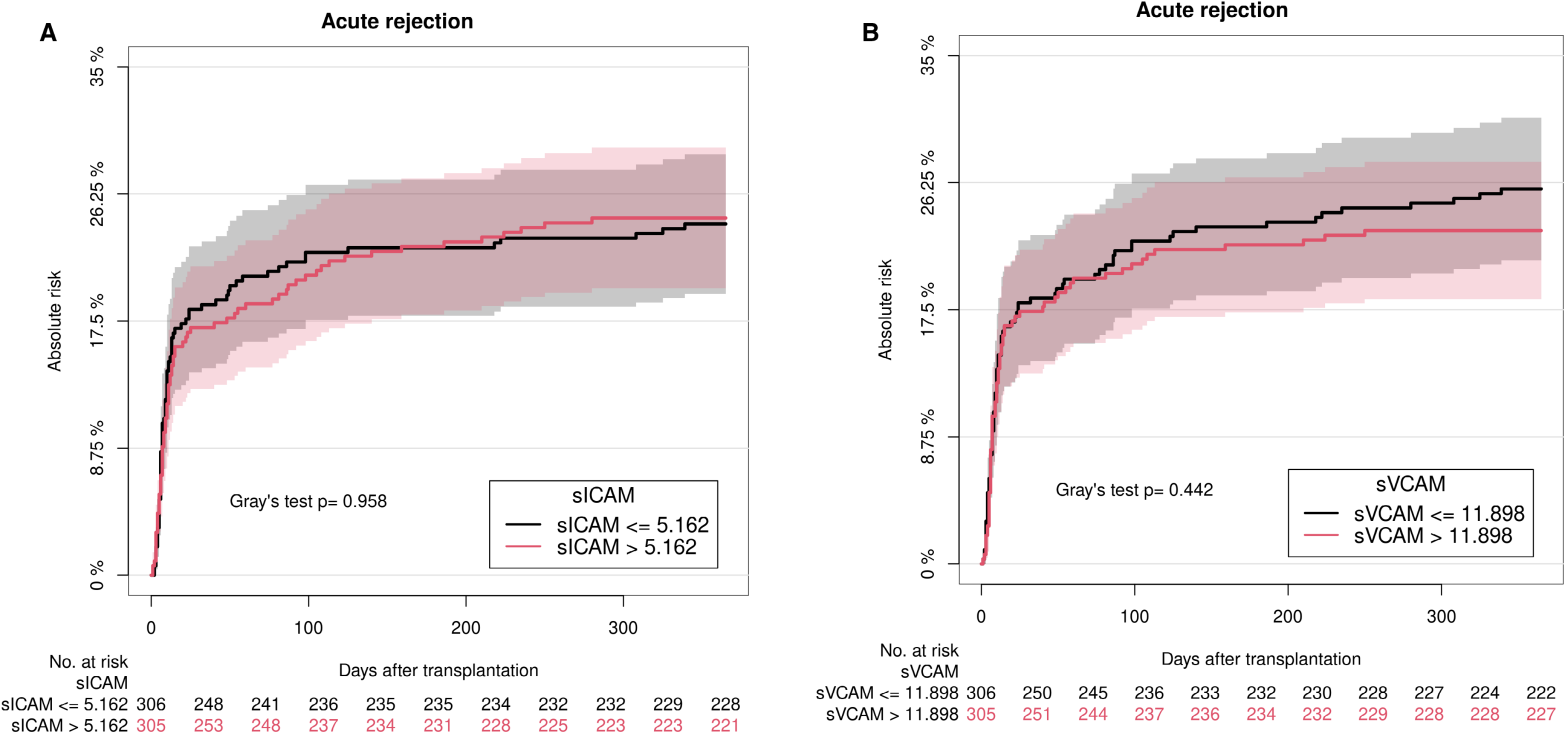
Curves of cumulative incidence for acute rejection within the first year after transplantation in recipients with low and high levels of endothelial cell activation markers at the time of transplantation. Curves of cumulative incidence for acute rejection within the first year after transplantation and endothelial cell activation markers: sICAM‐1 (A) and sVCAM‐1 (B). Recipients are divided into two groups based on the median level of markers: lowest 50% (black line) and highest 50% (red line). Death was treated as a competing risk in the analysis. Below the graphs are numbers at risk. 95% confidence intervals are shown. sICAM‐1: soluble intercellular adhesion molecule 1; sVCAM‐1: soluble vascular cell adhesion protein 1.

No associations were found between the endothelial cell activation markers and acute rejections within the first year of transplantation (Table 3C and Figure 3).

## 4 DISCUSSION

The main finding of the study was that serum levels of sICAM‐1 and sVCAM‐1 at the time of transplantation were robustly associated with all‐cause mortality in the years following transplantation. Additionally, we found that high levels of sICAM‐1 were associated with graft loss, especially in patients without preformed DSA that are antibodies directed towards non‐self HLA molecules in the donor.

Consistent with the findings in our study regarding the association between vascular inflammation in the transplant candidate and mortality after transplantation, it has been reported that kidney transplanted recipients with severe arteriosclerosis in native vessels show increased severity of allograft arteriosclerosis, decreased allograft survival and increased mortality in parallel.^6^ Moreover, a recipient phenotype characterized by accelerated atherosclerosis has been associated with the development of de novo DSA.^6^ In our study the importance of pretransplant biomarkers and preformed DSA was the focus and, unfortunately, we do not have data on the development of de novo DSA. Consequently, we cannot evaluate if a phenotype defined by vascular inflammation also promotes de novo DSA in our study cohort. DM, MACE and predialysis might, beside age, sex and DSA, in theory be important confounders or perhaps intermediate factors in associations between vascular inflammation and all‐cause mortality in our study. We found that vascular inflammation was associated with pretransplant dialysis but not with pre‐existing DM and only weakly associated with a history of MACE in the transplant candidate. Additionally, the association between sICAM‐1 and mortality after transplantation persisted after adjustment for these variables whereas the association was less robust regarding sVCAM‐1. These findings suggest a residual effect of vascular inflammation in relation to mortality most convincing for sICAM‐1. In accordance with this, it has previously been reported that vascular inflammation is also associated with mortality in patients with middle‐to‐high cardiovascular risk^24^ and in type 2 diabetic patients with microalbuminuria.^25^ Observations in these patient groups can, therefore, be extended also to kidney transplanted patients.

In our cohort, pretransplant serum levels of sICAM‐1 were associated with graft loss. Additionally, our data showed that vascular inflammation was associated with DGF, which is an early and well‐established risk factor in premature graft failure.^35^ This association was robust in recipients without preformed DSA, even after adjusting for confounders/intermediate factors already discussed above. However, we found no association between endothelial cell activation markers and acute rejections, which is another risk factor in graft loss.^35^ Therefore, data suggest that in the transplant candidate the pre‐existing global burden of vascular inflammation contributes to DGF, long‐term graft failure and mortality by other mechanisms than early acute rejection episodes. Consistent with this speculation, sICAM but not hsCRP, IL‐6 of TNF‐α, predict increased risk of progressive nephropathy in patients with type 1 diabetes^22^ and a comorbidity index strongly associated with cardiometabolic disease is associated with survival after kidney transplantation.^36^

In contrast to our findings, both sVCAM‐1 and sICAM‐1 were associated with the first episode of acute rejection and graft loss in a small study of 86 kidney transplanted recipients.^37^ The two studies differ in several variables. In the study by Perez et al.,^37^ acute rejection was registered throughout the entire follow‐up time with a mean of 5.4 years. In our study cohort, the outcome variable acute rejection was restricted to the first year of transplant when we consider this diagnosis to be best defined. Furthermore, Perez et al.^37^ divided endothelial cell activation markers into quartiles. It was only the lowest quartile compared to the higher quartiles that associated with lower rejection‐free survival. In contrast, we used endothelial cell activation markers as continuous variables in our study. We chose this approach because we consider it to be a more consistent variable when compared across studies, as a division into groups based on quantiles will be more dependent on the individual study cohort.

We found only statistically significant associations between endothelial cell activation markers and mortality, DGF or graft loss in recipients without preformed DSA. However, the analysis was not statistically significant in recipients with preformed DSA and there was no significant interaction between endothelial cell activation markers and DSA status except for sVCAM‐1 and DGF. Therefore, final conclusions cannot be made regarding the effect of vascular inflammation on the transplantation outcome in the recipient group with preformed DSA present. A lower number of recipients with preformed DSA compared with the group size of recipients without preformed DSA is a likely explanation. Additionally, it is possible that preformed DSA is more harmful for the outcome of transplantation than vascular inflammation, which may consequently be of less clinical importance in recipients with preformed DSA. Preformed DSA was associated with acute rejection during the first year after transplantation and with graft loss especially during the first years after transplantation in accordance with consensus in the literature^4^

In our study cohort 25% of recipients were transplanted against DSA evaluated by Luminex single antigen beads but with negative CDC cross‐matching. This reflects the retrospective study design. Thus, in the inclusion period 2009‐2015 CDC assays, which detect high levels of cytotoxic DSA,^38^ were routinely used for HLA allo‐antibody screening and cross‐matching before transplantation in the study cohort. The newer Luminex microbead assays with higher sensitivity and specificity of HLA‐antibody detection^38^ were implemented later for routinely evaluation before transplantation. Thus, the detection of DSA by Luminex assays were performed retrospectively in our study and no supplemental immunosuppression was given to recipients with preformed DSA, which would be considered as a risk factor in the clinical practice of today. Since the beginning of kidney transplantation history, the presence of preformed DSA has been reported to be more common in patients with a higher frequency of allogenic HLA antibodies such as women with a history of pregnancy and previously transplanted patients with a history of rejection^39^ regardless of the immunological method used. In accordance with this, it was less than 50% of recipients with preformed non‐self HLA antibodies who had DSA and among recipients with DSA it was a risk factor to be a woman or a previously transplanted patient. We do not have information about pregnancies in our study cohort, but the presence of HLA alloantibodies detected with a Luminex screening assay have been reported in more than 50% of women after pregnancy.^40^ HLA immunization in previously transplanted patients will to some extent depend on the HLA matching between recipient and donor in the preceding transplantations.^4^, ^5^

We have in Table 4 summarized our findings of associations between biomarkers of vascular inflammation and systemic inflammation before transplantation, preformed DSA and outcome variables including acute rejection within the first year after transplantation, graft loss and all‐cause mortality after transplantation based on data in the present study as well as data from our first study of associations between systemic inflammation and outcomes in the same cohort of kidney transplanted patients.^30^ It emerges that preformed DSA are important in acute rejection and thereby in graft loss whereas vascular inflammation is important in graft loss and patient survival especially in patients without preformed DSA, suggesting other pathways. In our present study, endothelial cell activation markers were positively correlated with circulating levels of TNF‐α, IL‐6 and hsCRP at the time of transplantation. This association is plausible with the well‐established observation that proinflammatory cytokines such as TNF‐α induce endothelial cell activation and vascular inflammation.^9^, ^41^ We cannot rule out that biomarkers of vascular inflammation act as surrogate markers of systemic chronic inflammation, but we have previously reported that TNF‐α, IL‐6 and hsCRP are not associated with graft failure in our study cohort.^30^

**TABLE 4:** Summary of associations between inflammatory biomarkers, preformed DSA and outcomes after kidney transplantation in the study population.

	Systemic inflammation^a^	Vascular inflammation	Preformed DSA
	TNF‐α, IL‐6, IL‐10, hsCRP	sICAM‐1 and sVCAM‐1	
Delayed graft function	Yes	Yes	Yes
Acute rejection	No	No	Yes
Graft loss	No	Yes (sICAM‐1)	Yes
All‐cause mortality	Yes	Yes	No

^a^ Based on data in ref. 30. DSA, donor‐specific antibodies.

Limitations of the study should be considered. It was preferable if the inclusion period of transplanted patients had been extended beyond 2015. The inclusion period for the cohort in this study was thus from 1 January 2009 to 31 December 2015 and the follow‐up of the primary outcome all‐cause mortality and the secondary outcome graft loss were both updated to July 2020. This gave the study a median follow‐up time of 8.3 years (range 4.6‐11.5 years) that we consider to be a strength of the study. We could not extent the inclusion period further as this study was a retrospective cohort study with a biobank at the time of transplantation approved by the regional ethical authority in Denmark for the specific time. Moreover, the routinely performance of immunological assessment has been changed later with the implementation of Luminex‐based assays. As a result, fewer patients are transplanted against Luminex detected DSA today compared to the period 2009‐2015 and we cannot exclude that this would have strengthen the association between sICAM and the outcome graft loss as our results indicate the association to be strongest in recipients without DSA. Data on obesity, smoking, physical fitness and anti‐inflammatory medicine intake was not available in our study, and we cannot rule out that inflammatory markers in the study just reflect these factors.^42^, ^43^, ^44^ Induction therapies, immunosuppressive maintenance treatment and transplantation may change vascular inflammation levels. We did not have plasma available in our cohort to explore vascular inflammation biomarkers' trajectory after transplantation. It is possible that various immunosuppressive regiments affect associations between endothelial cell activation, graft loss and mortality. In our study cohort a standard immunosuppressive treatment was used for most patients and this hypothesis can, therefore, not be addressed. The inter‐assay CV was high in the kit used to measure soluble CAMS. In accordance with this, biomarkers in systemic low‐grade inflammation are known to be high in our laboratory both in Meso Scale assays^42^ as well as in high‐sensitivity ELISA assays.^45^ The serum samples were stored at −20°C prior to the analyses of soluble CAMs and measurements were all performed during the same period in the end of 2016 in this study. Frozen sample storage is known to cause protein degradation.^46^ The samples in this study were from patients and no internal control was implemented for time of storage. It is most likely that these methodological limitations decrease the potential for detecting associations between inflammatory biomarkers and clinical outcomes. At least hsCRP is a stable marker both in relation to storage and inter‐assay CV.^46^ Soluble CAMs were weakly correlated with hsCRP, IL‐6 and TNF‐α in this study and despite a potential limitation due to protein degradation and a high inter‐assay CV, sICAM‐1 and sVCAM‐1 showed still robust associations with all‐cause mortality and DGF in patients without DSA. In contrast to hsCRP, sICAM was also associated with graft loss. Finally, the study tested only associations and not causality

Data in our study points to vascular inflammation in the transplant candidate as a potential target for intervention to improve the outcome of transplantation. A recent experimental study with anti‐ICAM‐1 monoclonal antibodies in liver transplantation in five non‐human primates showed improved long‐term liver allograft survival compared to a traditional immunosuppressive regimen.^47^ This implies a pivotal effect of specific pharmacological targets against vascular inflammation in the transplant setting. In contrast, a European multicentre study has demonstrated a lack of efficacy of anti‐ICAM‐1 as an induction treatment during the first 6 days post‐operatively in relation to DGF, acute rejection within the first 3 months, graft survival and patient survival at year one after transplantation in an unselected population of cadaveric renal transplant recipients.^48^ Authors suggested that it was conceivable that the short prophylactic administration was not appropriate, and the efficacy of anti‐ICAM‐1 therapy might be worthwhile to re‐evaluate in a selected patient population.^48^ Our data support that anti‐ICAM‐1 therapy might be interesting to evaluate in relation to long‐tern graft survival as the primary endpoint rather than short‐term acute rejection and in a subpopulation of patients without preformed DSA and with high levels of vascular inflammation.

In conclusion, this study demonstrates that pre‐existing vascular inflammation in the recipient is a risk factor for graft loss and mortality after kidney transplantation, especially in the transplant candidate without preformed DSA. We suggest that biomarkers of vascular inflammation add to the risk estimation before kidney transplantation.

## AUTHOR CONTRIBUTIONS

KPL and HB developed the hypotheses of the study and they designed, coordinated and conducted the study and were main responsible for the writing of this paper. KPL established the clinical biobank and databases. KPL performed the statistical analyses, under the supervision of FE. FE performed all statistical model control and produced all figures. BKP housed analyses for the inflammatory markers. SSS was responsible for the clinical patient data. All authors contributed to the writing process and the scientific discussion of data.

## FUNDING INFORMATION

Funding was received from The Danish Kidney Society. The Centre for Physical Activity Research (CFAS) is supported by TrygFonden (grants ID 101390, ID 20045 and ID 125132). During the study period, the Centre of Inflammation and Metabolism (CIM) was supported by a grant from the Danish National Research Foundation (DNRF55).

## CONFLICT OF INTEREST

Helle Bruunsgaard has been immunological investigator in a project that received sponsored laboratory materials from One Lambda, Thermo Fischer, CA, USA. No commercially sponsored laboratory materials or assays have been used to analyse recipient serum samples in this study.

## Data Availability

The data that support the findings of this study are available on request from the corresponding author. The data are not publicly available due to privacy or ethical restrictions.
